# Lipids, lipid-modifying drug targets with venous thromboembolism: a mendelian randomization study

**DOI:** 10.1016/j.clinsp.2026.101163

**Published:** 2026-09-22

**Authors:** Liuyan Nie, Yongmei Han, Fenping Zheng

**Affiliations:** a Department of Rheumatology, Sir Run Run Shaw Hospital, Zhejiang University School of Medicine, Hangzhou, China; b Department of Endocrinology, Sir Run Run Shaw Hospital, Zhejiang University School of Medicine, Hangzhou, China

**Keywords:** Mendelian randomization, Venous thromboembolism, Lipids, Statins, eQTL

## Abstract

**Background:**

The role of lipid-lowering therapies (e.g., statins) during anticoagulation for Venous Thromboembolism (VTE) remains unclear. This study investigated the causal role of lipid traits in VTE and evaluated lipid-lowering drug targets using a Mendelian Randomization (MR) approach.

**Methods:**

Genetic variants associated with lipid traits and lipid-lowering drug targets were extracted from the Global Lipids Genetics Consortium GWAS. Summary statistics for VTE, Deep Vein Thrombosis (DVT), and Pulmonary Embolism (PE) were obtained from UK Biobank. For associated targets, expression quantitative trait loci data were analyzed using summary-data-based MR. Colocalization analysis assessed shared genetic signals, and molecular docking explored plausible ligand-target binding poses.

**Results:**

Higher triglyceride levels were associated with lower VTE (OR = 0.998, 95% CI: 0.996‒1.000, p = 0.035) and DVT risks (OR = 0.998, 95% CI: 0.997‒1.000, p = 0.012). Genetic mimicry of Lipoprotein Lipase (LPL) enhancement was nominally associated with lower VTE odds (OR = 0.997, 95% CI: 0.995‒1.000, p = 0.02) and with lower PE odds after Bonferroni correction (OR = 0.996, 95% CI: 0.994‒0.998, p = 5.29E-06), but not DVT. LPL expression in whole blood showed a significant MR association with PE (OR = 1.001, 95% CI: 1.000‒1.002, p = 0.039) and moderate colocalization (PP.H4 = 0.68). Molecular docking predicted plausible active-site or allosteric binding poses for three LPL agonists (binding energies ≤ -6.8 kcal/mol).

**Conclusions:**

Genetically proxied LPL enhancement was nominally associated with modestly lower composite VTE risk and was associated with lower PE risk after multiple-testing correction, although no association was observed for DVT. These findings support a potential role for LPL-related pathways in VTE biology and warrant independent genetic and functional validation before clinical translation.

## Highlights

• Drug-target MR identified LPL as a candidate pathway in VTE.
• Higher triglyceride levels were inversely associated with VTE risk.
• Genetically proxied LPL enhancement reduced VTE and PE risk.
• SMR and colocalization analyses provided supportive evidence.
• Docking analyses demonstrated stable interactions with LPL.

## Introduction

Venous Thromboembolism (VTE), which includes Deep Vein Thrombosis (DVT) and Pulmonary Embolism (PE), constitutes a substantial global health challenge. It is characterized by high morbidity and mortality, surpassed only by myocardial infarction and stroke.^1^ Following an initial VTE, patients remain at a high risk of recurrent thrombosis, and nearly half of survivors may experience long-term complications, such as pulmonary hypertension and post-thrombotic syndrome, which lead to a marked decline in patients’ quality of life and impose a significant financial burden on healthcare systems.^2^^,^^3^

Although anticoagulants, particularly Direct-acting Oral Anticoagulants (DOACs) are widely used as first-line therapies for both the treatment and prevention of VTE, the current paradigm with anticoagulants alone is not universally effective, and the increased risk of causing a clinically significant bleeding episode remains concerning.^4^, ^5^, ^6^, ^7^ Taken together, these observations highlight the need for a deeper understanding of unravel mechanism that contribute to VTE to identify novel therapeutic approaches.

Recent literatures have highlighted the intricate relationship between coagulation and inflammation, where coagulation cascade activation stimulates the immune system, and innate immune cells, in turn, promote thrombus formation ‒ a process known as immune-thrombosis.^8^ Consequently, drugs with anti-inflammatory properties, like statins, might be a potential option to decrease incident and recurrent VTE.^9^^,^^10^ Statins are 3-Hydroxy-3-Methylglutaryl-Coenzyme A Reductase (HMGCR) inhibitors, which are the most powerful cholesterol-lowering drugs and the best-studied drugs available. Data are available on primary^9^^,^^11^ and secondary prevention^10^ in statin users during anticoagulation for VTE, as well as bleeding^12^ and mortality rates.^13^ However, the mechanism is still not clear. Recent studies indicate that statins may help prevent VTE by exerting anti-inflammation effects, improving endothelial function and anti-thrombotic, independent of modifying lipid profile.^14^^,^^15^ The association between VTE and lipid-lowering therapies other than statins, such as Proprotein Convertase Subtilisin/Kexin type 9 (PCSK9) inhibitors, remains understudied and warrants further investigation. While Randomized Controlled Trials (RCTs) are the gold standard for assessing efficacy, large-scale RCTs are limited, and conducting them remains challenging due to the nonspecific nature of VTE symptoms,^16^ imaging tests are essential for diagnosis, but dynamically monitoring drug effects in individuals remains challenging.

Mendelian Randomization (MR) analysis offers a useful alternative to RCTs.^17^ MR is an analytical approach that employs genetic variants ‒ specifically Single Nucleotide Polymorphisms (SNPs) identified through Genome-Wide Association Studies (GWAS) ‒ as proxies to infer the causal relationship between exposures, such as risk factors or potential drug targets, and health outcomes.^18^^,^^19^ Drug-target MR can show the impact of modifying biomarkers through specific drug targets on long-term health outcomes, predict the efficacy of drugs, and uncover any adverse reactions mediated by specific targets.^18^ MR analysis can predict RCT outcomes,^20^^,^^21^ and has recently been widely applied in drug target development and repurposing for cardiovascular, neurological diseases, and cancers.^22^^,^^23^ Notably, recent genetic study using Mendelian Randomization (MR) have yielded conflicting conclusions regarding the causal role of specific lipid-modifying targets in VTE risk,^24^ highlighting the complexity of the relationship and the need for more refined, mechanism-informed analyses.

To address these knowledge gaps and reconcile conflicting evidence, we conducted a comprehensive, multi-modal MR study. In this study, we aimed to explore the possible effects of lipid metabolism and targets for current/investigational lipid-lowering medications on the risk of VTE.

## Methods

This study was conducted in accordance with the STROBE-MR (Strengthening the Reporting of Mendelian Randomization of Observational Studies in Epidemiology) guidelines (Table S1).^25^ Our study framework is outlined in Fig. 1 The analysis was based on publicly accessible summary-level data from Genome-Wide Association Studies (GWAS) and expression Quantitative Trait Loci (eQTL) datasets (Table S2).

**Fig. 1:**
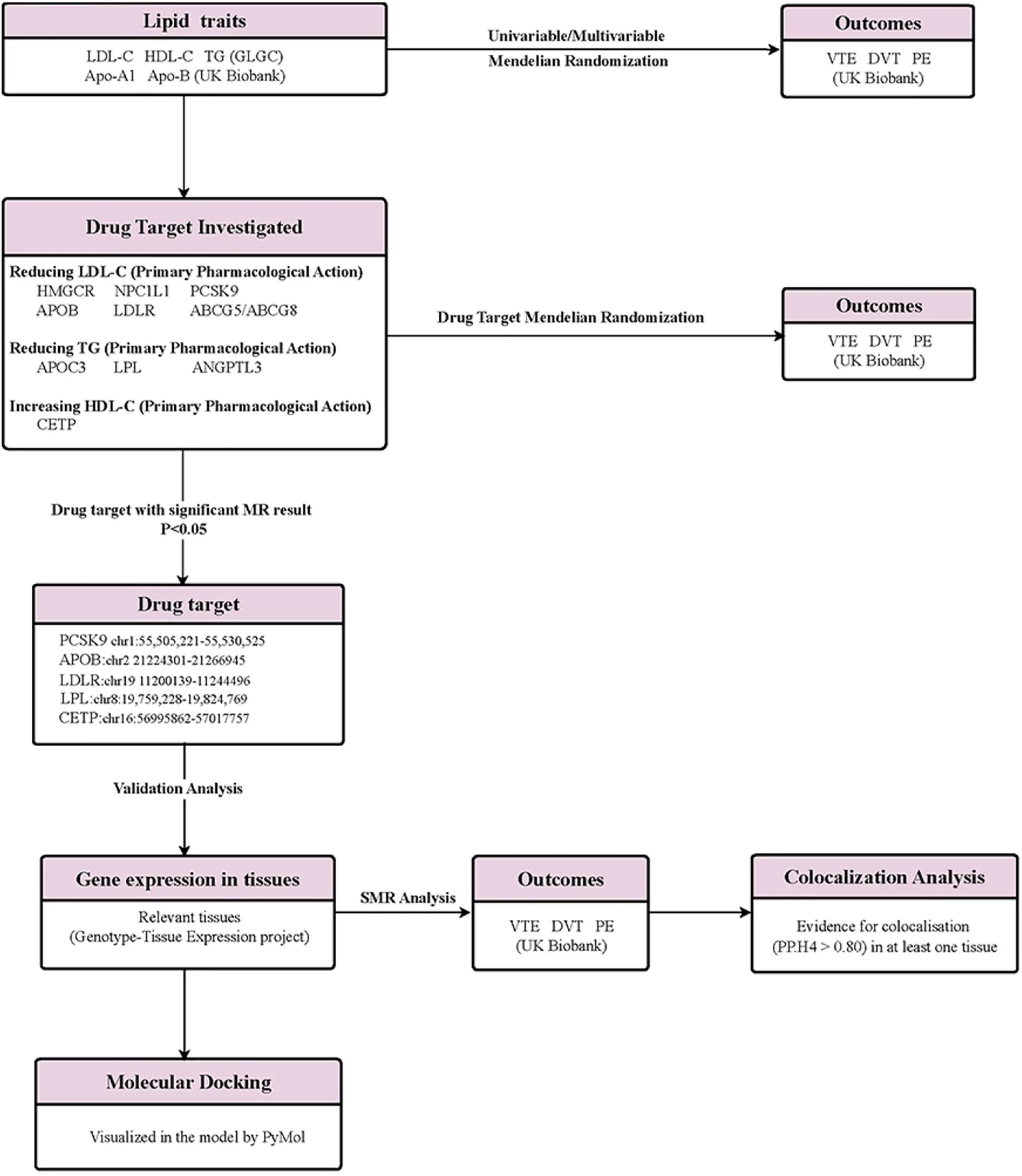
**Outline of the study design.** LDL-C, Low-Density Lipoprotein Cholesterol; TG, Triglyceride; HDL-C, High Density Lipoprotein Cholesterol; Apo-A1, Apolipoprotein-A1; Apo-B, Apolipoprotein-B; VTE, Venous Thromboembolism; DVT, Deep Vein Thrombosis; PE, Pulmonary Embolism; HMGCR, HMG-CoA Reductase; NPC1L1, Niemann-Pick C1-Like protein-1; PCSK9, Proprotein Convertase Subtilisin/Kexin type 9; ABCG5, ATP Binding Cassette Subfamily G Member-5; ABCG8, ATP Binding Cassette Subfamily G Member-8; APOB, Apolipoprotein B-100; LDLR, LDL Receptor; ANGPTL3, Angiopoietin-Like-3; APOC3, Apolipoprotein-C-III; LPL, Lipoprotein Lipase; CETP, Cholesteryl Ester Transfer Protein.

### Genetic instrumental variables selection

The Global Lipids Genetics Consortium GWAS meta-analysis screened for genetic variants that were significantly associated with Low-Density Lipoprotein (LDL-C), Triglycerides (TG), and High-Density Lipoprotein Cholesterol (HDL-C) (p < 5 × 10^−8^). These variants met the low linkage disequilibrium criteria (*r*^2^ < 0.001) and were separated by at least 10,000 kb.^26^ Similarly, instrumental variables for Apolipoprotein A1 (Apo-A1) and Apolipoprotein B (Apo-B) were obtained from the UK Biobank using the same selection criteria.

Recent dyslipidemia management protocols guide the selection of lipid-lowering agents,^27^ including statins, PCSK9 inhibitors, ezetimibe, mipomersen, fibrates, Angiopoietin-like 3 (ANGPTL3) inhibitors and antisense oligonucleotide targeting Apolipoprotein-C-III (APOC3) mRNA. Drug-related genes were identified through the DrugBank database (https://go.drugbank.com/) and associated publications.^28^, ^29^, ^30^ Based on the mode of action, these target genes were further divided into genes that lower LDL-C (i.e., LDL Receptor [LDLR], HMGCR, Niemann-Pick C1-Like Protein 1 [NPC1L1], PCSK9, Apolipoprotein B-100 [APOB], ATP-Binding Cassette subfamily G member-5 [ABCG5], and ATP-Binding Cassette subfamily G member 8 [ABCG8]), target genes that lower TG (i.e., Lipoprotein Lipase [LPL], peroxisome Proliferator-Activated Receptor Alpha [PPARA], ANGPTL3, and APOC3), and target genes that increase HDL-C (i.e., Cholesteryl Ester Transfer Protein [CETP]).

To proxy lipid modification through each drug target, we selected Single Nucleotide Polymorphisms (SNPs) located within ±100 kb of the corresponding gene that were associated with the relevant lipid trait at genome-wide significance (p < 5 × 10^−8^) in the Global Lipids Genetics Consortium GWAS meta-analysis, following a previously described approach.^26^ To maximize the power of the instrument for each drug target, the selected SNPs were allowed to be in weak linkage disequilibrium (*r*^2^ < 0.2, 250 kb). Since no significant SNPs were found within the PPARA locus, we excluded it from further analysis. Since the genes encoding ABCG5 and ABCG8 are located close to each other (ABCG5: chr2:44,039,611–44,066,004; ABCG8: chr2:44,066,103–44,105,605), we incorporated variants near these genes in our analysis. Ultimately, the following ten drug targets were included in this study: HMGCR, NPC1L1, PCSK9, APOB, ABCG5/ABCG8, LDLR, ANGPTL3, APOC3, CETP, and LPL.

For drug targets that showed an association with VTE, we also performed additional analysis using eQTL data from eQTLGen or GTEx Consortium V8, focusing on tissues where the target genes are highly expressed.^31^ eQTL data served as proxies for lipid-modulating drug target expression levels. This study focused exclusively on cis-eQTLs, which refer to genetic variants situated within 1 megabase of the corresponding target gene. This Summary-data-based MR (SMR) approach integrates GWAS and eQTL data to test for associations between the genetically predicted expression of a gene and the outcome.^32^

### Outcome

Pooled data on VTE (4620 cases and 356,574 controls), DVT (2116 cases and 359,078 controls) and PE (2118 cases and 359,076 controls) were obtained from UK Biobank-derived summary datasets in the IEU Open GWAS database (VTE: ukb-d-I9_VTE; DVT: ukb-d-I9_PHLETHROMBDVTLOW; PE: ukb-d-I26). UK Biobank is an extensive prospective cohort of >500,000 participants aged 40–69-years who were recruited between 2006 and 2010 for long-term health follow-up.^33^ The PE dataset used a main ICD-10 diagnosis of I26; VTE and DVT were analyzed according to the binary phenotype definitions supplied with their corresponding public releases. All datasets included participants of European ancestry. These publicly available case-control summary statistics do not provide event dates relative to UK Biobank enrollment; consequently, we could not restrict the analyses to incident-only events or distinguish incident from prevalent cases. The summary phenotypes also do not classify events as provoked or unprovoked.

### Statistical analysis

The Inverse-Variance Weighted (IVW) approach was applied to generate estimates of the causal effects of genetically proxied circulating lipid signatures and lipid-lowering drug targets on VTE. The reported odds ratios (ORs) for circulating lipid traits corresponded to a 1-SD genetically predicted increase in each trait. For drug-target MR, estimates were oriented to the expected lipid-modifying direction: a 1-SD decrease in LDL-C or TG, or a 1-SD increase in HDL-C, as appropriate.

Initially, univariable MR analyses were conducted to evaluate the potential associations between genetically predicted lipid profiles and VTE risk. Following this, the Multivariable MR (MVMR) methods was used to determine the independent effect of a lipid trait on VTE while adjusting for potential confounding or mediation by other lipid-related factors. For the MVMR analysis, we fitted a model l including TG, LDL-C, and Apo-B to determine which characteristic or characteristics contributed to VTE risk. We then adjusted for Apo-A1 and HDL-C in model 2 to clarify whether HDL-related traits have a causal impact on VTE development.

For a drug target that achieved significant VTE risk, the SMR method was further employed to assess the relationship between the expression of drug targets and VTE, utilizing summary data from eQTL and GWAS.^34^ Summary-data-based Mendelian Randomization (SMR) was performed using SMR software version 1.3.1 (https://cnsgenomics.com/software/smr). We used the default Heterogeneity in Dependent Instrument test (HEIDI) test threshold of p > 0.01 to indicate no significant heterogeneity due to linkage disequilibrium.^35^

Colocalization analysis assessed whether two traits share the same causal variant. In the text, we examined the Posterior Probability of Hypothesis 3 (PP.H3), in which both the drug targets and VTE were associated with the region by different variants, and Hypothesis 4 (PP.H4) that SNPs associated with the drug target and VTE are shared by the same causal variants. Here, proteins with strong colocalization support (PP.H4 > 0.8) were classified as tier 1 targets, those with moderate support (0.5 < PP.H4 < 0.8) as tier 2, and the rest as tier 3.^36^

An *F* statistic at least 10 was considered indicative of adequate instrument strength. To assess heterogeneity, we used Cochran's *Q*-test with a threshold of p < 0.05, indicating significant heterogeneity. If heterogeneity was detected, a random-effects model was used to estimate MR effect size. To assess horizontal pleiotropy, we performed the MR-Egger intercept test and MR-Pleiotropy Residual Sum and Outlier (MR-PRESSO) analysis. We also performed sensitivity analyses to test the robustness of our results. In addition, a leave-one-out method was used to test the effect of individual SNPs on the overall MR estimate. To validate significant MR associations, we applied stricter LD thresholds (*r*^2^ < 0.1, *r*^2^ < 0.01, and *r*^2^ < 0.001). Multiple testing was adjusted using Bonferroni correction: p < 0.01 (0.05/5) for five lipid traits and p < 0.005 (0.05/10) for ten drug targets. A two-sided p < 0.05 was considered statistically significant for other analyses.

All statistical analyses were conducted in R (version 4.1.2). The packages used included ‘Two Sample MR’, ‘Mendelian Randomization’, and ‘coloc’.

### Molecular docking

The crystal structure of human LPL (PDB ID: 6OAU) was obtained from the RCSB Protein Data Bank (https://www.rcsb.org/). Protein structure optimization was performed by adding polar hydrogen atoms and removing water molecules (except those coordinating the structural Ca²⁺). The three LPL agonists (50F10, C10d, NO-1886) were prepared using AutoDockTools 1.5.7 and converted into pdbqt format.^37^, ^38^, ^39^ Molecular docking was conducted with AutoDock Vina 1.2.2 to explore the conformational space and calculate binding affinities. The grid box was centered at (*x* = −21.7, *y* = 35.5, *z* = 21.5) with dimensions (*x* = 43.5, *y* = 79.3, *z* = 41.9) Å, and exhaustiveness was set to 32. The lowest-energy binding mode for each ligand was selected. Visualization of molecular docking results was performed with PyMOL 2.5 (3D) and LigPlot+ v2.2 (2D) to identify hydrogen bonds and hydrophobic interactions.

## Results

### Lipid traits and VTE risk

The instrumental variables corresponding to each lipid trait were summarized separately. Tables S3–S7 list the variables for LDL-C, TG, HDL-C, Apo-A1, and Apo-B, respectively. Among the five lipids, we found that TG was inversely related to the risk of VTE (OR = 0.998 [95% Confidence Interval ‒ 95% CI 0.996‒1.000], p = 0.035) and DVT (OR = 0.998 [95% CI 0.997‒1.000], p = 0.012). Similarly, higher levels of genetically predicted Apo-B (OR = 0.999 [95% CI 0.998‒1.000], p = 0.038) was related to a lower risk of DVT. No meaningful relationships were detected between other lipid traits and VTE risk (Fig. 2). Scatter plots of association between lipid traits and VTE were presented in Figure S1. Significant heterogeneity was found between the five lipid traits and VTE, but no horizontal pleiotropy was detected (Tables S8 and S9). The phenotype-specific pattern was not uniform: genetically predicted higher TG was inversely associated with the composite VTE phenotype and DVT, whereas no statistically significant association was observed for PE.

**Fig. 2:**
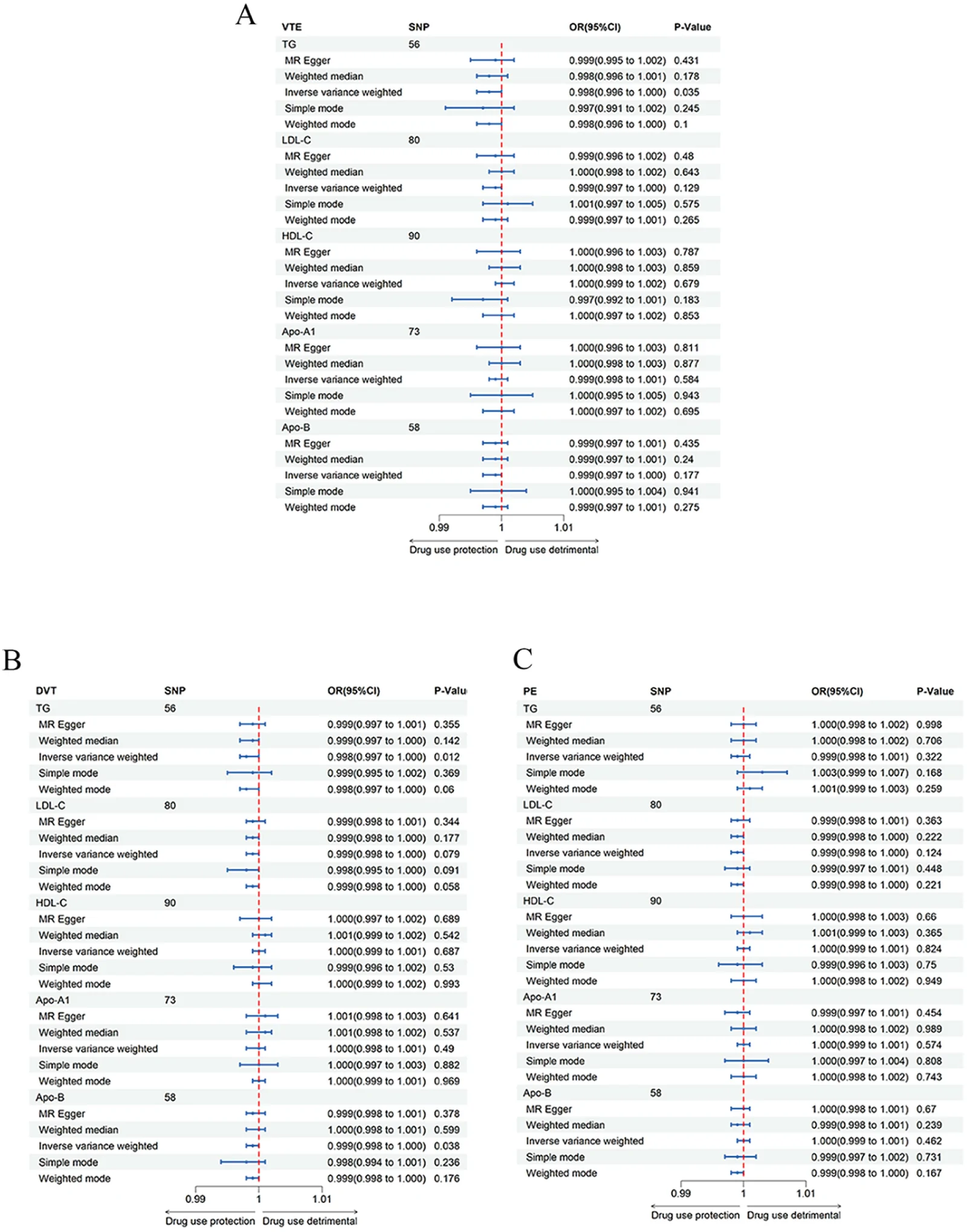
**Univariable Mendelian randomization results using different methods.** (A) Venous thromboembolism; (B) Deep vein thrombosis; (C) Pulmonary embolism. SNP, Single Nucleotide Polymorphisms; OR, Odds Ratio; CI, Confidence Interval; LDL-C, Low-Density Lipoprotein Cholesterol; TG, Triglycerides; HDL-C, High-Density Lipoprotein Cholesterol; Apo-A1, Apolipoprotein-A1; Apo-B, Apolipoprotein-B; VTE, Venous Thromboembolism; DVT, Deep Vein Thrombosis; PE, Pulmonary Embolism.

When LDL-C, TG, and Apo-B were assessed together in multivariable MR method, just TG kept a solid, potentially causal relationship with VTE (OR = 0.997 [95% CI 0.994‒1.000], p = 0.030) and DVT (OR = 0.998 [95% CI 0.996‒1.000], p = 0.012). In a multivariable MR analysis that included Apo-A1 and HDL-C, neither was distinguishable from the null, which is consistent with the results of univariable MR (Fig. 3).

**Fig. 3:**
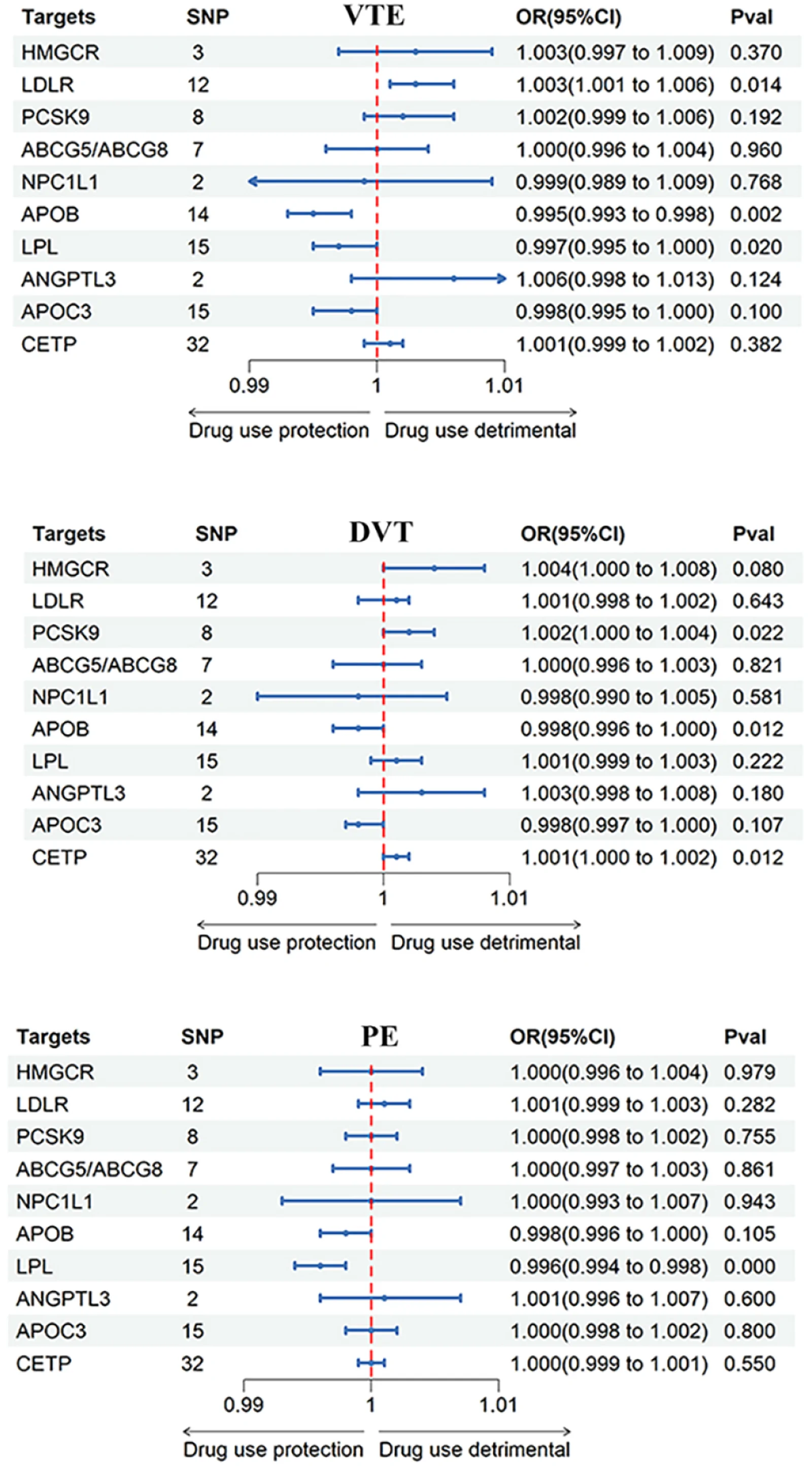
**Multivariable Mendelian randomization using the inverse-variance weighted method.** Model 1 included apolipoprotein-B, low-density lipoprotein cholesterol and triglycerides. Model 2 included apolipoprotein-A1 and high-density lipoprotein cholesterol. SNP, Single Nucleotide Polymorphisms; OR, Odds Ratio; CI, Confidence Interval; LDL-C, Low-Density Lipoprotein Cholesterol; TG, Triglycerides; HDL-C, High-Density Lipoprotein Cholesterol; Apo-A1, Apolipoprotein-A1; Apo-B, Apolipoprotein-B; VTE, Venous Thromboembolism; DVT, Deep Vein Thrombosis; PE, Pulmonary Embolism.

### Lipid-modifying drug targets and VTE risk

Table S10 listed the instrumental variables related to lipid-modified drug targets, including HMGCR, NPC1L1, PCSK9, APOB, ABCG5/ABCG8, LDLR, ANGPTL3, APOC3, CETP, and LPL. The associations between genetic proxies for these ten lipid-lowering agents and the risk of VTE are summarized in Fig. 4 and Table S11. LPL enhancement, linked to a 1-SD reduction in TG, was nominally associated with lower odds of VTE (OR = 0.997, 95% CI: 0.995‒1.000, p = 0.02) and was associated with lower odds of PE after Bonferroni correction (OR = 0.996, 95% CI: 0.994‒0.998, p = 5.29E-06). Similarly, APOB inhibition was protective and reduced VTE (OR = 0.995, 95% CI: 0.993‒0.998, p = 0.002) and DVT risks (OR = 0.998, 95% CI: 0.996‒1.000, p = 0.012). In contrast, LDLR inhibition, PCSK9 inhibition, CETP inhibition were found to be suggestively associated with higher risk of VTE. No statistically significant relationship was found between other genetic mimicries of drug targets (HMGCR, NPC1L1, ANGPTL3, APOC3 and ABCG5/ABCG8) and VTE risk. The MR-Egger intercept tests (all p > 0.05) did not detect evidence of directional horizontal pleiotropy (Table S12). In addition, the results remained robust in the leave-one-out sensitivity analysis (Fig. 5). Further analyses using more stringent LD thresholds (*r*^2^ < 0.1, *r*^2^ < 0.01, and *r*^2^ < 0.001) showed minimal impact on confidence interval widths, except for LDLR and CETP (Tables S13–S17). For LPL enhancement, the inverse association was observed for the composite VTE phenotype and PE but not for DVT. Thus, neither the TG nor the LPL signal was consistent across both VTE subtypes. The absence of a significant MR-Egger intercept does not exclude residual horizontal pleiotropy.

**Fig. 4:**
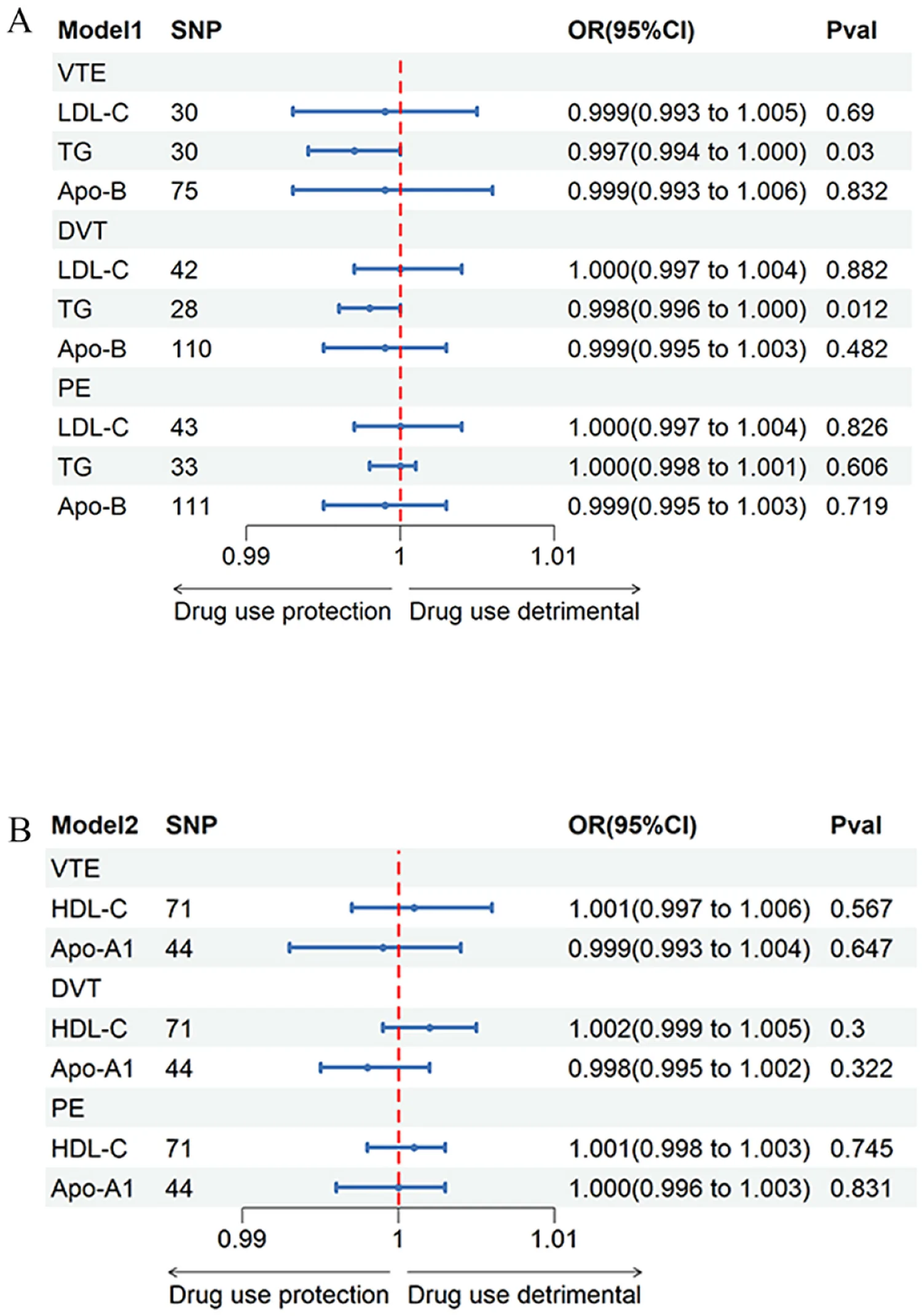
**Association of genetically proxied drug targets with risk of venous thromboembolism.** SNP, Single-Nucleotide Polymorphisms; OR, Odds Ratio; CI, Confidence Interval; HMGCR, HMG-CoA Reductase; NPC1L1, Niemann-Pick C1-Like protein-1; PCSK9, Proprotein Convertase Subtilisin/Kexin type-9; APOB, Apolipoprotein B-100; ABCG5, ATP Binding Cassette Subfamily G Member-5; ABCG8, ATP Binding Cassette Subfamily G Member-8; LDLR, LDL Receptor; ANGPTL3, Angiopoietin-Like-3; APOC3, Apolipoprotein-C-III; LPL, Lipoprotein Lipase; CETP, Cholesteryl Ester Transfer Protein; VTE, Venous Thromboembolism; DVT, Deep Vein Thrombosis; PE, Pulmonary Embolism.

**Fig. 5:**
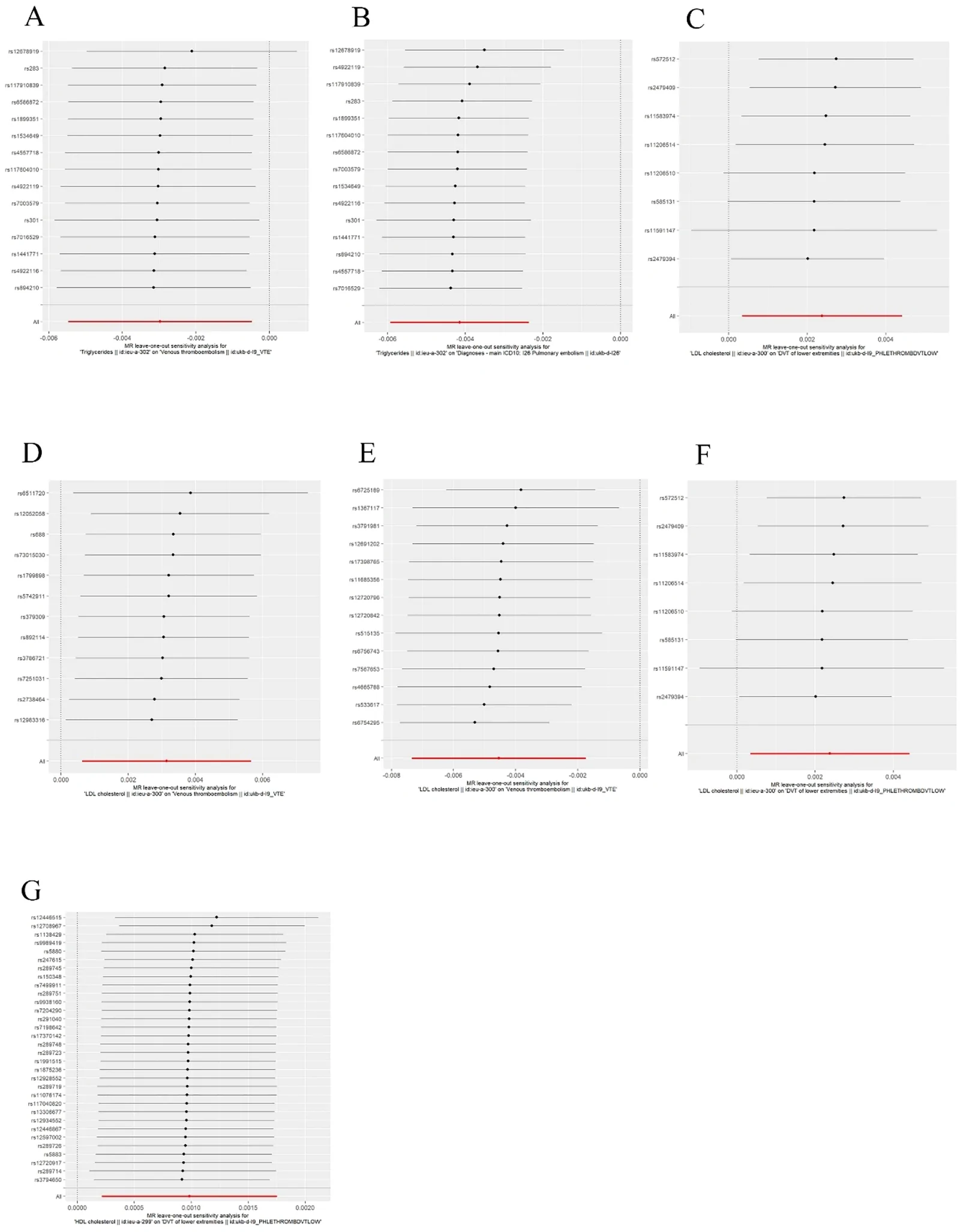
**Leave-one-out plots of MR analysis results for five significant drug targets with venous thromboembolism.** (A) LPL → VTE; (B) LPL → PE; (C) PCSK9 → DVT; (D) APOB → VTE; (E) APOB → VTE; (F) LDLR → DVT; (G) CETP → DVT.

### Gene expression and VTE risk

SMR analysis provided suggestive evidence that increased LPL gene expression in whole blood (equivalent to a one-SD increase) was related to a higher risk of VTE (OR = 1.001, 95% CI: 1.000‒1.002, p = 0.039) and PE (OR = 1.001, 95% CI: 1.000‒1.002, p = 0.003). No significant associations were found in the expression of PCSK9, LDLR, CETP, APOB and VTE outcomes (Table S18). The HEIDI test for LPL expression in whole blood gave p = 0.887 (VTE) and p = 0.458 (PE), confirming that the observed SMR associations are unlikely to be driven by linkage disequilibrium. For other gene-outcome pairs, HEIDI p‑values were also > 0.05, except for CETP where the test could not be performed due to an insufficient number of independent instruments (Table S18).

Furthermore, colocalization analysis was performed to assess the possibility that gene expression-associated variants and VTE-associated variants in relevant tissues were derived from the same causal SNPs. Moderate colocalization evidence was observed for LPL expression in whole blood and PE (PP.H4 = 0.68), classifying it as a tier 2 target. Other gene targets with weaker colocalization evidence were categorized as tier 3 (Table S19).

To explore potential ligand-LPL interactions, molecular docking was performed between three investigational LPL agonists (50F10, C10d, NO-1886) and the human LPL crystal structure (PDB: 6OAU). All three compounds yielded energetically favorable predicted poses (50F10: −8.3; NO-1886: −6.9; C10d: −6.8 kcal/mol) (Table S20). Analysis of binding modes revealed distinct interaction patterns (Fig. 6, Table S20). 50F10 formed two hydrogen bonds with catalytic triad residues Ser132 (2.8 Å) and His241 (3. Å), suggesting active‑site binding. In contrast, C10d and NO‑1886 bound at allosteric sites near the lid domain, each forming a single hydrogen bond (Arg243 for C10d, Glu179 for NO‑1886) and multiple hydrophobic contacts. None of the compounds interfered with the structural Ca^2+^ ion. These predicted binding modes provide structural hypotheses for ligand-LPL interactions. Further biochemical and functional studies are required to determine whether these interactions enhance LPL activity and influence VTE-related pathways.

**Fig. 6:**
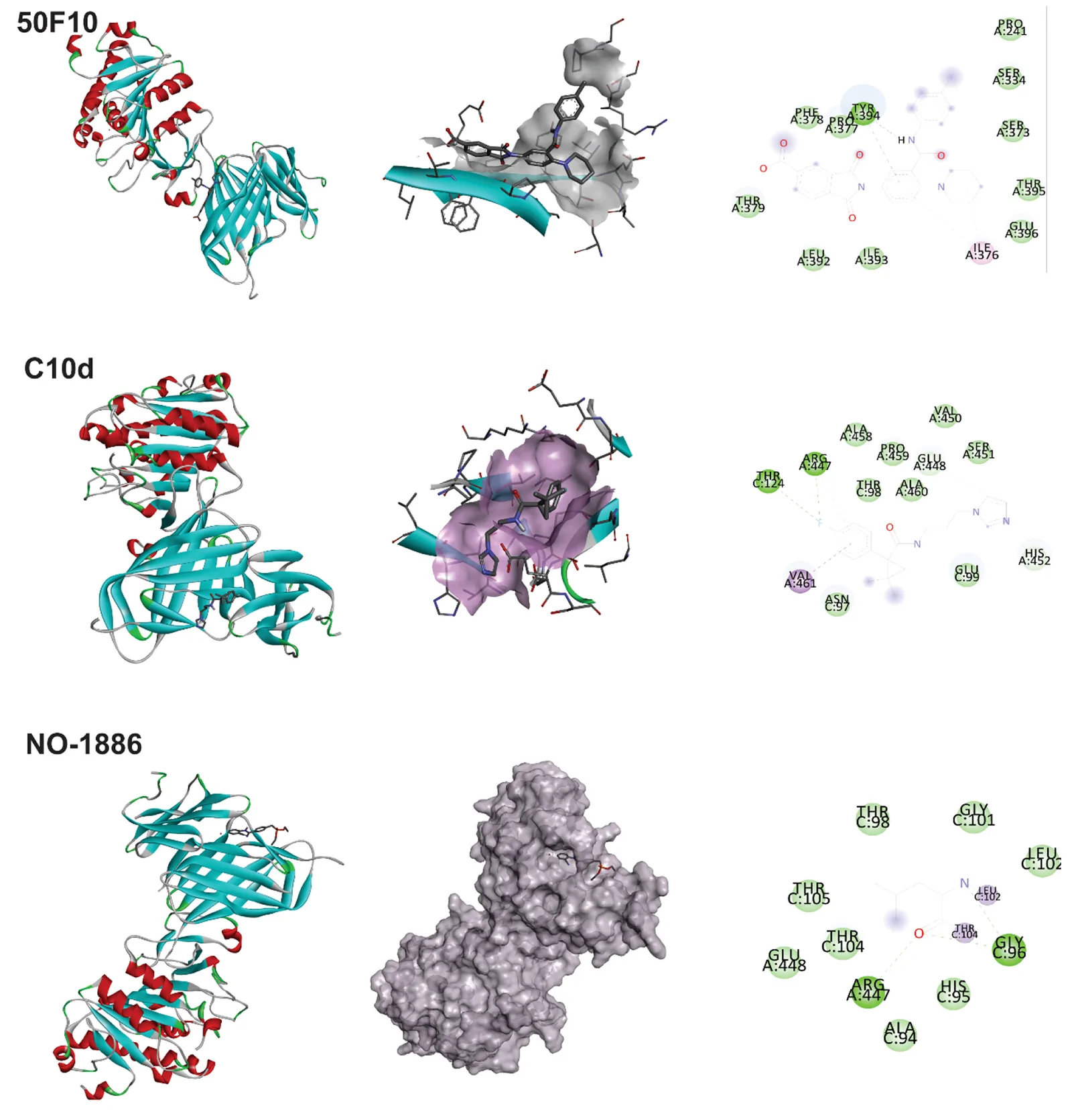
**Predicted binding poses of LPL agonists**. 50F10 (A), C10d (B), and NO-1886 (C) were docked to the human LPL structure (PDB ID: 6OAU). Left: overall binding pose; middle: binding-pocket view; right: two-dimensional interaction diagram.

## Discussion

This investigation sheds new light on the potential associations among lipid profiles, therapeutic targets involved in lipid regulation, and VTE risk. While previous Mendelian Randomization (MR) studies have established associations between circulating lipid traits and VTE,^24^ our study advances the field by dissecting the specific causal roles of lipid-lowering drug targets. A modest inverse association between genetic variants mimicking LPL enhancement and composite VTE was observed, although it did not meet the Bonferroni-corrected threshold for drug-target analyses. The direction of association was consistent across multiple genetic instrument specifications. Nevertheless, these analyses identify a small genetic signal and cannot establish that pharmacological LPL modulation would prevent VTE.

The LPL estimates were very small (OR = 0.997 for VTE and OR = 0.996 for PE per genetically proxied 1-SD reduction in TG), corresponding to only 0.3%‒0.4% lower relative odds. At an illustrative baseline risk of 1%, these estimates would equate to approximately 0.03‒0.04 fewer events per 1000 individuals, substantially smaller than the risk gradients associated with established VTE factors such as active cancer, major surgery, immobility, or prior VTE.^1^^,^^2^ Given differences in study design and exposure scale, this comparison is contextual only, and the MR estimates should not be interpreted as evidence of clinical utility or as predictions of drug effects.

The drug-target and expression analyses yielded different directions of association: variants mimicking LPL enhancement were associated with slightly lower VTE odds, whereas genetically predicted higher LPL expression in whole blood was associated with slightly higher VTE odds. Several explanations are possible. LPL expression in circulating immune cells may capture inflammatory signaling rather than systemic enzymatic activity, and expression may be compensatory in metabolic or inflammatory states.^40^^,^^41^ Differences between gene expression and protein activity, tissue context, non-linear effects, and residual pleiotropy may also contribute. These explanations are post hoc and remain speculative.

Compared with other lipid regulatory targets, the unique location of LPL is highlighted. We found that there was no significant association between VTE risk and genetic inhibition of APRIC3 or ANGPTL3, which also reduced triglycerides. This contrast may reflect differences in instrument strength, target biology, pleiotropic pathways, or statistical power; it does not establish that LPL has a unique therapeutic benefit or that triglyceride reduction through other targets is irrelevant to VTE.

The therapeutic relevance of these findings remains uncertain. LPL modulation has been observed as a pharmacological property of several clinically used drugs, including statins, fenofibrate, metformin, thiazolidinediones, and omega-3 fatty acids, although it is not their principal mechanism of action.^42^ Direct LPL agonists, including NO-1886, C10d, and 50F10, remain investigational.^42^ Although the small MR effect sizes and lack of independent replication preclude conclusions regarding clinical benefit, our findings provide a genetic rationale for further experimental evaluation of LPL-related pathways in VTE. Further studies are required to determine whether pharmacological LPL modulation can produce clinically meaningful effects.

Molecular docking was used to examine whether the three investigational LPL agonists could adopt plausible binding poses in the LPL crystal structure (6OAU). The predicted active-site and allosteric interactions are consistent with physical binding hypotheses, but docking energies do not demonstrate target activation, antithrombotic activity, or clinical efficacy. These in silico results should therefore be regarded as hypothesis generating and require biochemical and functional validation.

Contrary to conventional expectation, our study found an inverse relationship between higher TG levels on VTE risk. Interestingly, MVMR analyses confirmed a direct causal link, independent of other lipid traits, aligning with prior MR studies.^43^^,^^44^ There are several possible explanations for this discrepancy. First, Mendelian Randomization (MR) estimates reflect lifelong genetic exposure, which may differ from short-term metabolic states captured in observational studies. Second, triglyceride-related genetic variations may affect fibrinolytic pathways (e.g., tPA activity) independently of procoagulant effects.^43^^,^^45^ Sample overlap between exposure and outcome datasets may bias MR estimates toward observational associations, particularly for weak instruments. Therefore, results involving Apo-A1 and Apo-B should be interpreted with caution. Third, the influence of selection bias, residual pleiotropic or competing biological pathways cannot be ruled out. Therefore, our findings should be interpreted with caution and not as direct evidence of clinical protection from elevated circulating triglyceride levels, rather, they highlight a complex biology that warrants further mechanistic investigation.

The subtype results further argue against a single uniform protective interpretation. Higher genetically predicted TG was associated with DVT but not PE, whereas genetically proxied LPL enhancement was associated with PE but not DVT. These differences may reflect distinct pathophysiology between DVT and PE, limited statistical power within the smaller subtype case sets, phenotype misclassification, residual pleiotropy, or chance in the context of multiple comparisons. Accordingly, the composite VTE findings should not be generalized to all VTE phenotypes.

This investigation has several limitations. First, MR infers lifelong effects of genetic predisposition, which may not directly mirror the effects of pharmacological interventions.^46^ Second, the odds ratios were extremely close to 1.00 and imply very small relative and illustrative absolute differences; therefore, statistical significance should not be equated with prognostic or therapeutic relevance. Third, the subtype pattern was inconsistent: TG was associated with DVT but not PE, whereas LPL enhancement was associated with PE but not DVT. Fourth, the UK Biobank summary phenotypes are binary case-control outcomes without event dates, so we could not restrict the analysis to incident cases or separate incident from prevalent events. They also do not distinguish provoked from unprovoked VTE, creating clinical heterogeneity that may obscure subtype-specific mechanisms. Fifth, no independent validation cohort was available. We attempted replication in FinnGen and MGI, but the required variants could not be assembled into valid instrument sets; this unsuccessful attempt does not constitute replication and substantially limits generalizability. Partial sample overlap between the Apo-A1/Apo-B exposure data and UK Biobank outcomes may also introduce bias, despite the estimated overlap-related bias being small. Sixth, residual horizontal pleiotropy, selection bias, competing pathways, and chance cannot be excluded by the available sensitivity analyses. Finally, molecular docking is an in-silico prediction that does not capture LPL dynamics in a cellular membrane environment and cannot establish agonist activity or VTE prevention. Independent genetic cohorts, phenotype definitions that separate incident, provoked, and unprovoked events, and biochemical and in vivo experiments are required before clinical translation.

## Conclusions

In conclusion, genetically proxied LPL enhancement was nominally associated with modestly lower odds of composite VTE and was associated with lower odds of PE after multiple-testing correction, but no association was observed for DVT. The integrated genetic analyses suggest that LPL-related pathways may contribute to VTE biology, although the small effect sizes, subtype differences, and lack of independent replication limit clinical interpretation. These findings provide a rationale for independent replication and functional studies to determine whether LPL modulation has therapeutic relevance.

## Data and materials

The original contributions presented in the study are included in the Article/Supplementary Material, further inquiries can be directed to the corresponding author.

## Consent to publish declaration

Not applicable.

## Ethics and consent to participate declarations

Not applicable.

## Funding

This work was supported by Grant No. Y202351295 from Scientific Research Fund of Zhejiang Provincial Education Department and 2023-KYY-518053-0090 from 10.13039/501100004835Zhejiang University.

## Conflicts of interest

The authors declare that the research was conducted in the absence of any commercial or financial relationships that could be construed as a potential conflict of interest.

## Data Availability

The authors confirm that the data supporting the finding of this study are available within the article and its Supplementary Material.
