# Supplementary material for: Lipids, lipid-modifying drug targets with venous thromboembolism: a mendelian randomization study
Source: Clinics (Sao Paulo). 2026 Sep 22;81:101163. doi: 10.1016/j.clinsp.2026.101163 (PMC13691490; doi:10.1016/j.clinsp.2026.101163)
Supplement: Supplementary file 1 [file mmc1.docx]

**Supplementary Material**

Table S1	STROBE-MR checklist of recommended items to address in reports of Mendelian randomisation studies

Table S2	Information of GWAS and eQTLsummary data

Table S3	Genetic variants that were used as instruments for low-density lipoprotein cholesterol

Table S4	Genetic variants that were used as instruments for triglyceride

Table S5	Genetic variants that were used as instruments for high-density lipoprotein cholesterol

Table S6	Genetic variants that were used as instruments for Apolipoprotein A1

Table S7	Genetic variants that were used as instruments for apolipoprotein B

Table S8	Association between genetically-predicted lipid traits and the risk of venous thromboembolism

Table S9	Heterogeneity and pleiotropy tests of instrument effects (lipid traits)

Table S10	Characteristics of lipid-lowering genetics variants in target genes

Table S11	Association of genetically proxied lipid-lowering drug targets with risk of venous thromboembolism

Table S12	Heterogeneity and pleiotropy tests of instrument effects (drug targets)

Table S13	Association of genetic mimicry of LPL enhancement with risk of VTE accounting for LD structure (r2<0·2, r2<0·1, r2<0·01, r2<0·001)

Table S14	Association of genetic mimicry of PCSK9 enhancement with risk of DVT accounting for LD structure (r2<0·2, r2<0·1, r2<0·01, r2<0·001)

Table S15	Association of genetic mimicry of APOB enhancement with risk of VTE accounting for LD structure (r2<0·2, r2<0·1, r2<0·01, r2<0·001)

Table S16	Association of genetic mimicry of LDLR enhancement with risk of VTE accounting for LD structure (r2<0·2, r2<0·1, r2<0·01, r2<0·001)

Table S17	Association of genetic mimicry of CETP inhibition with risk of DVT accounting for LD structure (r2<0·2, r2<0·1, r2<0·01, r2<0·001)

Table S18	Association between genetically-predicted expression in whole blood or tissues of identified targets and VTE/DVT/PE in the SMR analysis

Table S19	Colocalisation results

Table S20	Molecular docking interactions of LPL agonists (50F10, C10d, NO‑1886) with human LPL (PDB: 6OAU)

**Supplementary Figure S1**
